# Analytical Performance Characteristics of the Cepheid GeneXpert Ebola Assay for the Detection of Ebola Virus

**DOI:** 10.1371/journal.pone.0142216

**Published:** 2015-11-12

**Authors:** Benjamin A. Pinsky, Malaya K. Sahoo, Johanna Sandlund, Marika Kleman, Medha Kulkarni, Per Grufman, Malin Nygren, Robert Kwiatkowski, Ellen Jo Baron, Fred Tenover, Blake Denison, Russell Higuchi, Reuel Van Atta, Neil Reginald Beer, Alda Celena Carrillo, Pejman Naraghi-Arani, Chad E. Mire, Charlene Ranadheera, Allen Grolla, Nina Lagerqvist, David H. Persing

**Affiliations:** 1 Department of Pathology, Stanford University School of Medicine, Stanford, California, United States of America; 2 Department of Medicine, Division of Infectious Diseases and Geographic Medicine, Stanford University School of Medicine, Stanford, California, United States of America; 3 Clinical Microbiology Laboratory, Stanford Health Care and Stanford Children’s Health, Palo Alto, California, United States of America; 4 Cepheid, Solna, Sweden; 5 Cepheid, Sunnyvale, California, United States of America; 6 Lawrence Livermore National Laboratory, Livermore, California, United States of America; 7 Department of Microbiology and Immunology, University of Texas Medical Branch, Galveston, Texas, United States of America; 8 Special Pathogens Program, National Microbiology Laboratory, Public Health Agency of Canada, Winnipeg, Manitoba, Canada; 9 The Public Health Agency of Sweden, Solna, Sweden; Division of Clinical Research, UNITED STATES

## Abstract

**Background:**

The recently developed Xpert® Ebola Assay is a novel nucleic acid amplification test for simplified detection of Ebola virus (EBOV) in whole blood and buccal swab samples. The assay targets sequences in two EBOV genes, lowering the risk for new variants to escape detection in the test. The objective of this report is to present analytical characteristics of the Xpert® Ebola Assay on whole blood samples.

**Methods and Findings:**

This study evaluated the assay’s analytical sensitivity, analytical specificity, inclusivity and exclusivity performance in whole blood specimens. EBOV RNA, inactivated EBOV, and infectious EBOV were used as targets. The dynamic range of the assay, the inactivation of virus, and specimen stability were also evaluated. The lower limit of detection (LoD) for the assay using inactivated virus was estimated to be 73 copies/mL (95% CI: 51–97 copies/mL). The LoD for infectious virus was estimated to be 1 plaque-forming unit/mL, and for RNA to be 232 copies/mL (95% CI 163–302 copies/mL). The assay correctly identified five different Ebola viruses, Yambuku-Mayinga, Makona-C07, Yambuku-Ecran, Gabon-Ilembe, and Kikwit-956210, and correctly excluded all non-EBOV isolates tested. The conditions used by Xpert® Ebola for inactivation of infectious virus reduced EBOV titer by ≥6 logs.

**Conclusion:**

In summary, we found the Xpert® Ebola Assay to have high analytical sensitivity and specificity for the detection of EBOV in whole blood. It offers ease of use, fast turnaround time, and remote monitoring. The test has an efficient viral inactivation protocol, fulfills inclusivity and exclusivity criteria, and has specimen stability characteristics consistent with the need for decentralized testing. The simplicity of the assay should enable testing in a wide variety of laboratory settings, including remote laboratories that are not capable of performing highly complex nucleic acid amplification tests, and during outbreaks where time to detection is critical.

## Introduction

Ebolaviruses cause severe hemorrhagic fever with high mortality rates [1]. There are five known ebolaviruses: Zaire, Sudan, Bundibugyo, Reston, and Taï Forest [2]. Ebola virus (EBOV), the sole member of the species *Zaire ebolavirus*, caused the recent outbreak centered in West Africa [3].

EBOV is an enveloped, single-stranded, negative-sense RNA virus belonging to the *Filoviridae* family. The genome is approximately 19 kilobases in length, and contains seven genes: nucleoprotein (NP), viral protein (VP) 35, VP40, glycoprotein (GP), VP30, VP24, and RNA polymerase (L) [4].

Viremia is present when a patient is symptomatic and viral concentrations rise during the first week of illness [5], although cases of asymptomatic viremia have been reported [6]. In patients who survive, viremia usually becomes undetectable by the end of the second week of illness. Viral concentrations are inversely correlated with the outcome of disease [5]. Virus has been detected by nucleic acid amplification tests in saliva, stool, semen, breast milk, tears, nasal blood, and skin surface swabs [7, 8].

Reverse transcriptase (RT)-PCR and antigen detection are the most useful diagnostic tools considering the viremia pattern and the varying occurrence of antibody development [8–10]. Among the described EBOV PCR assays, performance varies and many tests fail to fulfill basic sensitivity requirements [11]. During the current outbreak in West Africa, the turnaround time for test results is up to 72 hours [8] for patients without local access to testing, adding to the risk of prolonging the epidemic by failing to stop transmission and delaying supportive care. The need for a sensitive and timely point-of-care test for EBOV is urgent.

The Xpert® Ebola Assay (Cepheid, Sunnyvale, CA, USA) is a novel assay for detection of EBOV from EDTA whole blood obtained by venipuncture, peripheral blood derived from finger-stick, and saliva obtained from buccal swabs. Xpert® is a fully automated RT-PCR system that integrates processing, amplification, and detection, and provides a result in 100 minutes from sample acquisition. Additionally, the system has remote monitoring and electronic reporting capability. This report describes the analytical performance characteristics of the Xpert® Ebola Assay on whole blood samples.

## Materials and Methods

### Xpert® Ebola Assay

For sample preparation, 0.1 mL whole blood or a swab to which whole blood has been allowed to absorb is transferred to the 2.5 mL Xpert® Ebola Sample Reagent (SR) bottle, which contains 4.5 M guanidinium thiocyanate. SR inactivates EBOV within the recommended incubation time and temperature (20 minutes at ambient temperature), denatures and liquefies the sample, stabilizes the nucleic acid target, and creates the salt concentration needed for subsequent RNA purification.

The SR buffer is transferred to the Xpert® Ebola Assay cartridge, where all steps of sample preparation, reverse transcription and multiplex, fluorescent real-time PCR occur [12]. All reagents required for these steps are included in the cartridge, either in liquid form or in lyophilized beads. Nucleic acids are isolated and purified, concentrated onto a small glass fiber column integrated into the base of the cartridge, and washed and eluted prior to combining with the RT-PCR reagents. The Sample Processing Control (SPC), included in the cartridge, is automatically spiked into the sample during processing to monitor target recovery and inhibition. Once the sample is loaded, the cartridge is closed and remains closed for the remainder of the assay including disposal of the cartridge. This helps prevent cross-contamination of amplicons in the laboratory. The cartridge is loaded into the GeneXpert® module where processing, detection, and fluorescent readout are performed automatically. Time to result is 100 minutes. Additionally, the GeneXpert® Ebola Assay system has remote monitoring and electronic reporting capability when the included computer has Internet access.

### Primer and Probe Design

The primers and probes in the Xpert® Ebola Assay detect two conserved regions within the glycoprotein (GP) and the nucleoprotein (NP) of the EBOV genome. The amplified target sequences are detected with TaqMan probes, each labeled with distinct proprietary fluorophores designed to operate with different detection channels of the optics of the GeneXpert system. Primers and probes for the EBOV targets are indicated in Table 1.

**Table 1:** Primer and probe sequences for the EBOV targets used in the Xpert® Ebola Assay.

Target	Primer/Probe	Sequence
GP	Forward primer	GGG CTG AAA ACT GCT ACA ATC TTG AAA TC
	Reverse primer	GGA AGC CCC GAA TCC CGT
	Probe	CCT GAC GGG AGT GAG TGT CTA CC
NP	Forward primer	GCT CCT TTC GCC CGA CTT TTG AA
	Reverse primer	CTG TGG CGA CTC CGA GTG CAA
	Probe	TGA GCA TGG TCT TTT CCC TCA AC
HMBS	Forward primer	CTG GCC TGC AGT TTG AAA TCA GTG
	Reverse primer	GCG GGA CGG GCT TTA GCT A
	Probe	TGG AAG CTA ATG GGA AGC CCA GTA CC

GP, Glycoprotien; NP, Nucleoprotein; HMBS, human hydroxymethilbilane synthase

The Xpert® Ebola Assay cartridge also includes two internal controls, the Sample Adequacy Control (SAC) and the SPC. The SAC detects a human genomic DNA target in the human hydroxymethylbilane synthase gene (HMBS). The proprietary SPC is an exogenous control that consists of armored RNA containing an artificial RNA sequence that does not exist in nature. The SPC amplicon shows no homology to the EBOV target sequences.

### Ethics, Whole Blood, Viral Stocks, and Concentration Estimates

De-identified EDTA-whole blood collected from healthy blood donors was purchased from the Stanford Blood Center (Palo Alto, CA, USA), Puget Sound Blood Center (Seattle, WA, USA), and Blodtappen, Karolinska University Hospital (Stockholm, Sweden). The *in vitro* investigational use of de-identified human blood products as diluent is not subject to Institutional Review Board (IRB) approval from these institutions, and neither written nor verbal consent was required for the use of these de-identified products in this study.

#### Inactivated EBOV Yambuku-Mayinga

This reagent was obtained through BEI Resources, National Institute for Allergy and Infectious Diseases (NIAID), National Institute of Health (NIH): Zaire Ebolavirus, Mayinga, Gamma-Irradiated, NR-31807, Lot 60428483 (EBOV Yambuku-Mayinga, EBOV/Yam-May. The stock concentration was value assigned using nCounter® Viral RNA Quantitation. The nCounter® Virus Assay Panel was previously designed by Lawrence Livermore National Laboratory (Livermore, CA, USA) and developed by nanoString® Technologies (Seattle, WA, USA) to detect and quantitate viral genomic RNA in nucleic acid samples. The panel consists of probes for 560 viral genomes, 26 of which detect regions of the EBOV genome, and six internal reference genes. For this work, each test was conducted on 5uL of total EBOV Yam-May inactive virus RNA that were prepared for hybridization, detection, and scanning following the manufacturer’s protocol. Samples were analyzed using the nCounter® Analysis System (nanoString® Technologies).

The raw data was normalized to five genes within the panel with the lowest coefficient of variation using nanoString's nSolver® software. The normalized results were expressed as total RNA counts per probe. Total RNA counts represent total instances of the viral genome present in a sample and were used to quantify copies of molecules per sample. The maximum count of any selected sample indicated the maximum molecules hybridized and detected by the nCounter® Analysis System. Thus, maximum count divided by total volume of added sample represent molecules present in the sample. For additional information, see S1 File. Dilutions of stock virus were made with pooled whole blood before testing. Spiked whole blood was centrifuged to obtain plasma for testing.

#### Infectious EBOV Makona-C07

Ebola virus/H.sapiens-tc/GIN/2014/Makona-C07 (species *Zaire ebolavirus*) (GenBank: KJ660347.2) was propagated on Vero E6 cell cultures, passage 3 (Galveston National Laboratory, University of Texas Medical Branch, Galveston, TX, USA). The stock concentration was assigned a value of 4.6x10^7^ PFU/ml using a plaque-forming assay on Vero E6 cells [13]. Dilutions of stock virus were made with pooled whole blood before testing.

#### EBOV Yambuku-Mayinga RNA

The RNA used originated from one unique specimen from a cell culture isolate obtained from the Public Health Agency of Sweden. The RNA stock was assigned a concentration based on its crossing threshold (Ct) values (EBOV GP 17.6 cycles, EBOV NP 17.4 cycles) against Ct values determined using a calibration curve composed of dilutions of standard RNA (Biosynthesis Inc., Lewisville, TX, USA) of known concentrations. The standard RNA concentration was specified in the Certificate of Analysis and verified by the Public Health Agency of Sweden using micro volume spectrophotometry. EBOV/Yam-May RNA was spiked into SR and then combined with whole blood prior to testing.

### Linearity/Dynamic Range

The results of the Xpert® Ebola Assay were compared to the results of the Altona RealStar® Ebolavirus RT-PCR Kit 1.0 (Altona Diagnostics, Hamburg, Germany) and BioFire Defense LLC FilmArray Biothreat-E Test® (BioFire Diagnostics, Salt Lake City, UT, USA), both of which target the L gene. Whereas the Xpert® Ebola Assay and BioFire Defense LLC FilmArray Biothreat-E Test® detect only EBOV, the Altona RealStar® Ebolavirus RT-PCR Kit 1.0 detects the five species in the *Ebolavirus* genus, including EBOV. All three assays have been issued an Emergency Use Authorization (EUA) by the U.S. Food and Drug Administration (FDA) [14]. The Altona RealStar® is approved for detection of virus in plasma and the FilmArray Biothreat-E Test® for detection in whole blood and urine. The Altona test was performed on a Rotor-Gene Q (Qiagen, Frederick, MD, USA). QIAamp® Viral RNA Mini Kit was used for manual RNA extraction from plasma. BioFire is a qualitative test and no Ct values are reported.

Inactivated EBOV/Yam-May was spiked into EDTA-whole blood and tested in replicates of three at seven (Xpert® Ebola Assay, and Altona RealStar® Ebolavirus RT-PCR Kit 1.0) or five (BioFire Defense LLC FilmArray Biothreat-E Test®) different concentrations from 1 to 1x10^6^ copies/mL whole blood. The correlation between EBOV concentration and Ct values for gene targets and IC, respectively, was estimated for Xpert® and Altona RealStar®. Negative controls, positive controls, and negative blood or plasma controls were run each day on each instrument.

### Limit of Detection/Analytical Sensitivity

Limit of Detection **(**LoD) testing was performed on inactivated virus, infectious virus, and RNA, as follows.

#### Inactivated EBOV

Inactivated EBOV/Yam-May (isolate Ebola virus/H.sapiens-tc/COD/1976/Yambuku-Mayinga, obtained through BEI Resources, NIAID, NIH) was spiked into EDTA-whole blood, and testing was performed on a 2-fold dilution series with 20 replicates at each concentration. The estimated LoD, the lowest concentration of sample that can be reproducibly distinguished from negative samples with 95% confidence or the lowest concentration at which 19 of 20 replicates are positive, was determined by Probit logistic regression. Testing was performed over 3 days using one reagent lot.

#### Infectious EBOV

EBOV Makona-C07 (Mak-C07) (Galveston National Laboratory, University of Texas Medical Branch, Galveston, TX, USA) was spiked into EDTA-whole blood and testing was performed on a 2-fold dilution series (50–12.5 PFU/mL) and 10-fold dilutions series (1–0.01 PFU/mL) on 6 dilutions in replicates of 4 on one reagent lot. The lowest dilution for which all replicates were detected was taken as the preliminary LoD. Verification of the preliminary LoD was performed on one reagent lot with 20 replicates. The LoD of active virus is reported as the lowest concentration of plaque-forming unit (PFU) per mL EDTA-whole blood at which at least 19 out of 20 replicates are positive.

#### EBOV RNA

EBOV/Yam-May (Ebola virus/H.sapiens-tc/COD/1976/Yambuku-Mayinga) RNA (Public Health Agency of Sweden) was spiked into EDTA-whole blood mixed with SR. Six dilutions were tested in replicates of 20 per dilution on one reagent lot. The LoD was estimated using Probit analysis. The claimed LoD was confirmed by analyzing at least 20 replicates on three reagent lots.

### Inactivation/Virucidal Efficacy

The efficiency of the Xpert® Ebola Assay SR to inactivate EBOV was determined with infectious EBOV/Mak-C07. In order to maximize the quantity of virus tested, the virus was not spiked into whole blood, but 0.1 mL at a concentration of 4.6x10^7^ PFU/mL was added directly to 2.5 mL SR and incubated at room temperature for 20 minutes. Prior to the addition of the EBOV/SR mixture to the cell culture, 0.5 mL of the EBOV/SR mixture was subjected to dialysis using a single-use Rapid Equilibrium Dialysis (RED) device in order to remove cell-toxic salts. The virucidal efficiency of the SR was determined by adding 150 μL of the dialysate to each of 3 wells in a 6-well tissue culture plate (well diameter 34.8 mm) containing Vero E6 cells. The cells were incubated for 1 hour at 37°C to allow virus adsorption and were then re-fed. Cytopathic effect (CPE) was monitored over 2 passages (7 days of incubation each). If any virus CPE was observed, a plaque-forming assay was used to verify and quantify any remaining infectious virus in the media. Positive controls included infectious virus added to the cell medium. Dialyzed SR alone in cell medium served as a negative control. A virus sample (infectious EBOV Kikwit, ~1.0x10^7^ PFU/mL) diluted 1:6 in AVL buffer (Qiagen, Frederick, MD, USA) and incubated for 10 minutes at ambient temperature was included as a sample inactivation control.

### Inclusivity and Exclusivity

#### Inclusivity

The analytical reactivity (inclusivity) of the Xpert® Ebola Assay was determined by testing four EBOV isolates/variants other than Mayinga that were available in the form of infectious EBOV or EBOV RNA: EBOV Makona (virus), Yambuku-Ecran (virus, GenBank KM655246.1) [15], Gabon (virus, GenBank KC242800.1), and Kikwit (RNA, GenBank KR867676.1). In addition, *in silico* analysis of all other available sequences of EBOV was performed; see S2 File.

The test samples were prepared and spiked into EDTA-whole blood, or if genomic viral RNA was used, into EDTA-whole blood mixed with SR. The final concentrations for testing (in PFU/mL [virus] or copies/mL [RNA]) were 1x LoD for Makona, whereas the final concentration of Ecran and Gabon was 3x LoD, as estimated from a preliminary value assignment using the plaque-forming assay on Vero E6 cells. The quantitative value of Kikwit RNA was determined spectrophotometrically, and the final testing concentration was approximately 5x LoD as estimated from titration of the Ct values of a dilution panel. Each test specimen was tested in replicates of 20.

#### Exclusivity

To evaluate analytical specificity, or possible cross-reaction or interference of other organisms in the detection of EBOV RNA using the Xpert® Ebola Assay, a number of viruses, bacteria, and parasites were tested at clinically relevant levels. If exclusivity organisms and viruses were not available, *in silico* analysis was performed; see S3 File. Specimens were prepared by spiking into EDTA-whole blood, or if genomic RNA/DNA of the organism was used, into EDTA-whole blood mixed with SR. Each sample was tested in replicates of three. If a positive Ct was detected for any organism in the panel, the testing was repeated with a lower concentration of the organism, and the highest concentration that would not produce false positive results was recorded. If the organisms were not available at the desired concentration, the testing was performed at the highest available concentration.

For both inclusivity and exclusivity testing, a negative control sample consisting of an EBOV negative EDTA-whole blood specimen was run in parallel with the test specimens. Each specimen as well as the negative control was tested in replicates of three using one lot of Xpert® Ebola Assays. One positive and one negative external control were run every day of testing. The positive control was comprised of EBOV GP and NP RNA at ~10x LoD (SeraCare Life Science, Gaithersburg, MD, USA) and the negative control was comprised of human DNA (hDNA) in TE buffer.

### Specimen Stability

To establish specimen transport and storage claims for the Xpert® Ebola Assay once the sample has been added to the SR, specimens were stored for various periods of time (up to 72 h) at different temperatures (5–45°C). The specimens were EBOV/Yam-Mayinga RNA at a level of 3 to 5x LoD, spiked into SR and then mixed with 0.1 mL EDTA-whole blood. Five replicates were tested at each condition except at time zero, where eight replicates were tested. Statistical significance of specimen stability at the conditions tested was determined by comparing Ct values of the test samples to the Ct values of the control sample (time zero) using one-way ANOVA. Dunnett’s multiple comparison method was also performed for the time points of each temperature compared to the control. Both the Ebola GP target Ct and the Ebola NP target Ct values were included in the analyses. One positive and one negative external control were run every day of testing.

## Results

### Linearity/Dynamic Range

**Fig 1:**
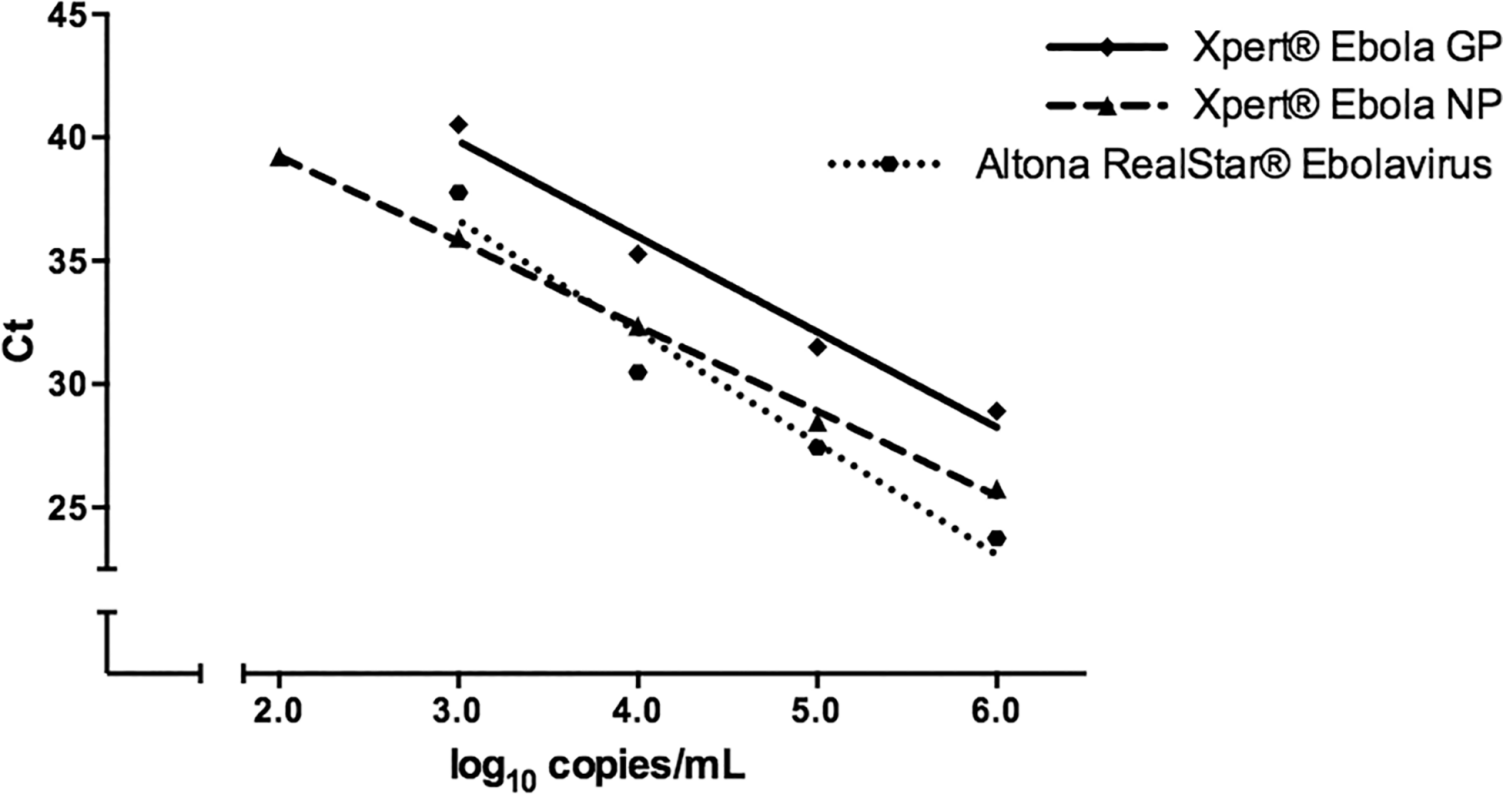
Linearity of the Xpert® Ebola Assay and Altona RealStar® Ebolavirus RT-PCR Kit 1.0. Serial dilutions of inactivated EBOV Yambuku-Mayinga was spiked into EDTA-whole blood and tested in triplicate. Linear regression lines are shown for the Xpert® Ebola nucleoprotein (NP) and glycoprotein (GP) targets (NP: y = -3.44x+46.11, R^2^ = 0.98; GP: y = -3.87x+51.45, R^2^ = 0.87), and Altona RealStar® Ebolavirus (y = -4.51x+50.17, R^2^ = 0.93).

Xpert was compared to the Altona RealStar® Ebolavirus RT-PCR Kit 1.0, and BioFire Defense LLC FilmArray Biothreat-E Test® using 5–7 serial dilutions of inactivated EBOV/Yam-May (Table 2). Since the BioFire test does not provide Ct values, correlations between viral concentration and Cts for targets were estimated for Xpert and Altona only (Fig 1). The response (Ct) of both EBOV targets of the Xpert assay (NP & GP) is linear with the log of input viral genome copies over 4–5 logs dilution, as is that of the Altona assay. The response of the ICs is constant, as is expected.

**Table 2:** Range finding analysis of the Xpert® Ebola Assay, Altona RealStar® Ebolavirus RT-PCR Kit 1.0, and BioFire Defense LLC FilmArray Biothreat-E Test® using Inactivated EBOV Yambuku-Mayinga.

Nominal copies per mL	No. of positive replicates /total replicates				
	Xpert			Altona	BioFire
	GP or NP	GP	NP		
1 x 10^6^	3/3	3/3	3/3	3/3	3/3
1 x 10^5^	3/3	3/3	3/3	3/3	3/3
1 x 10^4^	3/3	3/3	3/3	3/3	3/3
1 x 10^3^	3/3	3/3	3/3	3/3	1/3
1 x 10^2^	3/3	0/3	3/3	0/3	0/3
1 x 10^1^	0/3	0/3	0/3	0/3	Not tested
1	0/3	0/3	0/3	0/3	Not tested

### Limit of Detection/Analytical Sensitivity

The LoD for the Xpert® Ebola Assay was assessed first using inactivated EBOV/Yam-May. The GeneXpert® Ebola Assay was compared directly to the Altona RealStar® Ebolavirus RT-PCR Kit 1.0 (Table 3 *)*. The LoD was estimated using Probit analysis, including the 5 lowest concentrations at which each assay detected virus. The LoD for the Xpert® Ebola Assay was estimated to be 73 copies/mL (95% CI: 51–97 copies/mL), and for the Altona RealStar® Ebolavirus RT-PCR Kit 1.0 LoD was estimated to be 509 copies/mL (95% CI: 411–722 copies/mL). BioFire detected 19/20 replicates of inactivated EBOV/Yam-May in a concentration of 4,850 copies/mL, but inconsistent detection was observed at lower levels. It was therefore not included in further, detailed LoD analyses.

**Table 3:** Limit of Detection of the Xpert® Ebola Assay and Altona RealStar® Ebolavirus RT-PCR Kit 1.0 using Inactivated EBOV Yambuku-Mayinga.

Assay	Nominal copies/mL	Total replicates (n)	Total positives (n)	Positivity rate (%)	Positive GP (n)	Positive NP (n)
Xpert	24250	20	20	100	20	20
	1516	20	20	100	20	20
	758	19*	19	100	18	19
	379	20	20	100	16	20
	189	20	20	100	16	20
	95	20	19	95	11	17
	47	20	15	75	4	14
	24	20	12	60	4	8
Altona	24250	20	20	100	NA	NA
	1516	20	20	100	NA	NA
	758	20	19	95	NA	NA
	379	20	17	85	NA	NA
	189	20	10	50	NA	NA
	95	20	1	5	NA	NA

* One replicate was removed due to a manufacturing error.

NA, Not Applicable

LoD for the Xpert® Ebola Assay was also assessed using infectious EBOV/Mak-C07. A preliminary LoD for the infectious EBOV specimen in EDTA-whole blood was estimated at 1.0 PFU/mL (Table 4). This was verified by testing 20 replicates at this concentration: all replicates were positive confirming that the LoD was at least as low as 1.0 PFU/mL. Finally, LoD was assessed using EBOV/Yam-May RNA (Public Health Agency of Sweden). The numbers of positive replicates per dilution level are presented in Table 4. Using Probit analysis, the estimated LoD for EBOV/Yam-May RNA in EDTA-whole blood was 232 copies/mL (95% CI 163–302 copies/mL). LoD was verified by analyzing at least 20 replicates at the estimated LoD in three reagent lots; 95% of the replicates were positive in one reagent lot, and 100% in two reagent lots.

**Table 4:** Numbers of positive replicates per level in the Xpert® Ebola Assay for EBOV Makona-C07, and for EBOV Yambuku-Mayinga RNA, both in EDTA-whole blood.

	Nominal PFU/mL	Total replicates (n)	Total positives (n)	Positivity rate (%)
EBOV	50	4	4	100
Makona-C07	25	4	4	100
	12.5	4	4	100
	1	4	4	100
	0.1	3	1	33
	0.01	4	0	0
EBOV	700	20	20	100
Yambuku- Mayinga RNA	300	20	20	100
	150	20	13	65
	75	20	12	60
	30	20	9	45
	15	20	5	25

### Inactivation/Virucidal Efficacy

No signs of EBOV CPE were observed in any of the three plates of Vero E6 cell cultures exposed to 4.6x10^7^ PFU/mL of EBOV/Mak-C07 virus in the Xpert® Ebola SR reagent following 7 days of culturing (passage 1). Supernatants were removed and used to inoculate new cells (passage 2) and observed for another 7 days. CPE suggestive of filovirus activity was observed on day 3 in the passage 2-culture in 1 of 3 plates. Material from the plate showing CPE was placed in the plaque-forming assay to verify and quantify the EBOV. Briefly, increasing 10-fold dilutions of the samples were adsorbed to Vero E6 monolayers in duplicate wells (200μL). No plaque-forming units were detected above the limit of detection of 25 PFU/mL. Inactivation of EBOV was determined to be complete starting with levels of EBOV ≥10^6^ PFU/mL.

Under the conditions used, no viral infectious effect was observed in the negative control cell cultures incubated in media with dialyzed Xpert® Ebola SR only. The negative control sample with EBOV in AVL buffer also failed to show any CPE, thus demonstrating that the standard procedure to inactivate EBOV reduced the virus infectivity by ≥ 6 logs. The positive control virus sample in medium (not inactivated) exhibited 100% CPE in wells by day 3 of passage 1 CPE and showed the expected virus infectivity at the concentration used.

### Inclusivity and Exclusivity

The analytical reactivity for the Xpert® Ebola Assay was 100% (20 of 20) for EBOV Makona, Yambuku-Ecran, Gabon, and Kikwit (Table 5). The Makona isolate from Guinea (EBOV/Mak-C07) was tested at estimated 1x LoD and the other strains at approximately 3 to 5x LoD. *In silico* analysis concluded that the EBOV NP and GP oligonucleotides completely match all EBOV sequences present in GenBank. In addition, Visual OMP and thermo profile robustness testing suggested at least a 3°C Tm (annealing temperature) margin, indicating that the assay could be robust to some primer/probe to target mismatch (data not shown).

**Table 5:** Analytical Reactivity and Specificity (Inclusivity and Exclusivity).

Organism	Source^a^	Specimen Type	Test concentration	Unit (per mL whole blood)	Total replicates (n)	Total positives (n)
**EBOV**						
Makona-C07	GenBank:KJ660347.2	Infectious Virus	1x LoD	NA	20	20
Yambuku-Ecran	GenBank:KM6552461.1	Infectious Virus	3x LoD	NA	20	20
Gabon-Ilembe	GenBank:KC242800.1	Infectious Virus	3x LoD	NA	20	20
Kikwit-956210	GenBank:KR867676.1	Nucleic Acids	5x LoD	NA	20	20
**Non-EBOV ebolaviruses**						
Ivory Coast virus	UTMB-Tesh^b^	Nucleic Acids	546^h^	ng/mL	3	0
Reston virus	UTMB-Tesh	Nucleic Acids	3.0x10^5^	PFU/mL	3	0
Sudan virus Boneface	NA	*In silico *	NA	NA	NA	NA
Sudan virus Gulu	NA	*In silico *	NA	NA	NA	NA
Bunidbugyo virus	NA	*In silico *	NA	NA	NA	NA
**Other viruses**						
Chikungunya virus 181/25	UTMB-Tesh	Nucleic Acids	2798^h^	ng/mL	3	0
Rift Valley Fever virus SA51	UTMB-Tesh	Infectious Virus	7.5x10^5^	PFU/mL	3	0
Crimean Congo Hemorrhagic Fever Dubai	UTMB-Tesh	Nucleic Acids	3.4x10^6^	PFU/mL	3	0
Dengue virus Type 2	UTMB-Tesh	Nucleic Acids	2.7x10^6^	PFU/mL	3	0
Yellow Fever virus OBS-6745	UTMB-Tesh	Nucleic Acids	1.0x10^6^	PFU/mL	3	0
Influenza virus A H9N2	NVSL^c^	Nucleic Acids	1.0x10^5^	PFU/mL	3	0
Lassa virus Pinneo	UTMB-Tesh	Nucleic Acids	5.7x10^3^	PFU/mL	3	0
Marburg virus Angola	UTMB-Tesh	Infectious virus	2.6x10^6^	PFU/mL	3	0
Marburg virus Musoke	GenBank:NC_001608	Infectious virus	5.0x10^4†^	PFU/mL	3	0
Marburg virus Musoke	UTMB-Tesh	Nucleic Acids	6.0x10^4^	PFU/mL	3	0
Marburg virus Ravn	UTMB-Tesh	Nucleic Acids	4.8x10^5^	PFU/mL	3	0
Adenovirus	NA	*In silico *	NA	NA	NA	NA
Enterovirus	NA	*In silico *	NA	NA	NA	NA
Influenza virus B	NA	*In silico *	NA	NA	NA	NA
Marburg virus Ci67	NA	*In silico *	NA	NA	NA	NA
Rotavirus	NA	*In silico *	NA	NA	NA	NA
Respiratory Syncytial virus	NA	*In silico *	NA	NA	NA	NA
**Bacteria**						
*Coxiella burnetti*	CDC^d^	Nucleic Acids	50	ng/mL	3	0
*Hemophilus influenzae*	ATCC 51907D	Nucleic Acids	50	ng/mL	3	0
*Pseudomonas aeruginosa*	ATCC 15442D-5	Nucleic Acids	50	ng/mL	3	0
*Rickettsia conorii*	UTMB-Bouyer^e^	Nucleic Acids	50	ng/mL	3	0
*Rickettsia prowazekii*	UTMB-Bouyer	Nucleic Acids	50	ng/mL	3	0
*Rickettsia typhi*	UTMB-Bouyer	Nucleic Acids	50	ng/mL	3	0
*Salmonella bongori*	ATCC 43975D-5	Nucleic Acids	50	ng/mL	3	0
*Salmonella typhi*	ATCC 700931D-5	Nucleic Acids	50	ng/mL	3	0
*Shigella flexneri Type 2*	ATCC 29903D-5	Nucleic Acids	50	ng/mL	3	0
*Streptococcus pneumoniae*	ATCC 33400D-5	Nucleic Acids	50	ng/mL	3	0
*Yersinia enterocolitica*	DOD^f^ YERS095	Nucleic Acids	50	ng/mL	3	0
*Yersinia pestis*	DOD YERS023	Nucleic Acids	50	ng/mL	3	0
*Borrelia recurrentis*	NA	*In silico *	NA	NA	NA	NA
*Leptospira* genus	NA	*In silico *	NA	NA	NA	NA
*Neisseria meningitidis*	NA	*In silico *	NA	NA	NA	NA
*Rickettsia africae*	NA	*In silico *	NA	NA	NA	NA
*Vibrio cholerae*	NA	*In silico *	NA	NA	NA	NA
**Parasites**						
*Plasmodium falciparum*	NA	*In silico *	NA	NA	NA	NA
*Plasmodium malariae*	NA	*In silico *	NA	NA	NA	NA
*Plasmodium ovale*	NA	*In silico *	NA	NA	NA	NA
*Plasmodium vivax*	NA	*In silico *	NA	NA	NA	NA
*Trypanosoma*	NA	*In silico *	NA	NA	NA	NA
**Other**						
Mosquito	UC Davis^g^	Nucleic Acids	50	ng/mL	3	0
Tick	UC Davis	Nucleic Acids	50	ng/mL	3	0

LoD, Limit of Detection; PFU, Plaque Forming Unit

^a^GenBank or ATCC number, if available

^b^Dr. Robert Tesh, University of Texas Medical Branch, Galveston, TX

^c^National Veterinary Service Laboratory, Ames, IA

^d^Centers for Disease Control and Prevention, Atlanta, GA

^e^Dr. Donald Bouyer, University of Texas Medical Branch, Galveston, TX

^f^Department of Defense, Critical Reagents Program

^g^University of California, Davis

^h^RNA concentration of the stock material

The results of exclusivity testing are shown in Table 5. The analytical specificity of the Xpert® Ebola Assay for the evaluated organisms was 100%. *In silico analysis* of analytical specificity concluded that the Xpert® Ebola primer and probe sequences should not yield false positive EBOV results with any of the exclusivity pathogens listed in Table 5. The results of the *in silico* study also predicted that the Xpert® Ebola control oligonucleotides (SAC and SPC) are specific for their respective targets (data not shown). The Xpert® Ebola and SAC oligonucleotides did not show any cross reactivity towards the synthetic SPC RNA construct included in the assay and no oligo-oligo interactions of the Xpert® Ebola oligonucleotides potentially giving rise to nonspecific signals were predicted.

### Specimen Stability

There was no statistically significant difference in Ct values of the GP and NP targets in the Xpert® Ebola Assay for specimens stored for 24, 48, and 72 h compared to control at 5°C (p = 0.784 and 0.595) and for specimens stored 24 and 48 h at 30°C (p = 0.777 and 0.369), or for 24 h at 35°C (p = 0.159 and 0.337). The individual Ct values of each specimen stored at different temperature are presented in S1 Fig.

There was no statistically significant difference in Ct values of the NP target for specimens stored for 24, 48, and 72 h compared to controls at 25°C (p = 0.208), whereas the Ct values for the GP target exhibited a statistically significant difference for specimens stored at 25°C for 24, 48 and 72 h compared to control (p = 0.015). However, the difference at 24 h was <1.0 Ct compared to control, and this shift is within the normal assay variation. Furthermore, Dunnett’s comparison showed that there was no statistically significant difference in specimens stored at 25°C for 48 h and 72 h compared to 0 h. In summary, the difference at 24 h is not considered practically significant. Limited number of replicates were tested, which may account for the apparent anomaly in stability at 25°C. There was a statistically significant difference in Ct values of the GP and NP targets for specimens stored at 45°C for 24h compared to control (p = 0.001 and 0.001). The average Ct differences are 1.18 Ct for GP and 1.76 Ct for NP compared to control.

In conclusion, data support storage of whole blood specimens in the Xpert® Ebola SR for at least up to 72 h at 2–28°C, for a maximum of 48 h at 30°C and 24 h at 35°C.

## Discussion

The ability to rapidly diagnose EBOV disease is critical for effectively segregating non-EBOV infected patients from EBOV-infected patients, to identify EBOV as a possible cause of death in a postmortem setting and to provide appropriate supportive care in EBOV outbreaks. Current diagnostics are dependent on centralized facilities with long turnaround times. Here, we report the analytical characteristics of a novel EBOV molecular diagnostic assay, suitable for decentralized testing, with a 100-minute turnaround time, with higher analytical sensitivity than two other EUA-approved tests, with a robust virus inactivation system, considerable specimen stability and with strong inclusivity and exclusivity test performance.

The Xpert® Ebola Assay is a qualitative test but provides Ct values as an approximate indicator of virus load. Generally, EBOV quantitation is not performed for clinical purposes, as the correlations of virus load at diagnosis with clinical outcomes or as a guide to clinical management are areas that require further investigation. However, Ct values may ultimately provide a tool for monitoring disease progression. The inclusion of a novel sample adequacy control (SAC) provides greater confidence in the interpretation of negative results, especially in settings where sample degradation, such as post-mortem testing, is possible. In addition, for specimen types such as buccal swabs where visual cues of specimen adequacy may be lacking, a negative SAC value may indicate the need to re-sample the patient.

The performance of currently available EBOV diagnostic methods varies. A recent study showed that among 24 laboratories with in-house PCR methods, only 78.6% succeeded in detecting EBOV-positive samples with 1.6x10^5^ viral gene copies/mL [11], suggesting significant differences in operator skill and in the platform technologies employed. The need to define performance standards for EBOV diagnostic tests is critical. We show in this report that the Xpert® Ebola Assay is a robust test that detects EBOV at very low levels and yields highly reproducible results. The 100-minute turnaround time for Xpert® Ebola Assay is shorter than the Altona RealStar® Ebolavirus RT-PCR Kit 1.0, which requires approximately 3 hours to perform a 4 test batch, including significant hands-on time for manual extraction (45 minutes) and PCR set-up (30 minutes). The BioFire Defense LLC FilmArray Biothreat-E Test® lacks off-board virus inactivation, which reduces the number of pipetting steps and contributes to a turnaround time that is shorter than the Xpert® Ebola Assay by approximately 30 minutes. However, multiple FilmArray instruments would likely be required for testing in an outbreak setting as each FilmArray can run only one test at a time, whereas the GeneXpert® instrument can run 4, 8, 16 or 80 tests simultaneously, depending on the model. Additionally, the GeneXpert® Ebola Assay system can integrate into a cloud-based remote monitoring and electronic reporting system, adding the potential to track outbreaks in real time. A recent pilot of this software was used to geolocate nearly 2.5 million TB and rifampin resistance results emanating from GeneXpert® systems being used in four countries (personal communication, Chinmay Sheth, Cepheid, CA, USA). This system is designed to work within areas with limited bandwidth and inconsistent connectivity, which are characteristic of Ebola-affected regions of West Africa.

There have been a number of EBOV diagnostic assays developed that target the L, NP, GP and VP24 genes [16, 17]. The Xpert® Ebola Assay targets both the NP and GP genes in order to minimize the inherent risk of evolving EBOV variants that escape detection. Analysis of 99 genomes from 78 Ebola patients in Sierra Leone indicated that the West African 2014 lineage diverged from Central African lineages around 2004. The substitution rate observed in Sierra Leone early in the outbreak was twice as high within the Guinea and Sierra Leone outbreaks as between outbreaks in the past. Non-synonymous mutations were observed, potentially creating a risk for viral adaptation, which could affect both drug development and diagnostic assays [18]. However, ongoing analysis of virus sequences obtained from an additional 232 patients in Sierra Leone revealed the effects of purifying selection on the reduction of non-synonymous mutations in the setting of a prolonged outbreak and suggested that the substitution rate was more similar to past outbreaks [19]. In a study of another 175 EBOV genomes from Sierra Leone, the virus was independently shown to be evolving at a rate similar to that observed between previous outbreaks, though the emergence of multiple novel lineages was identified, with variants dispersed throughout the genome [20]. By incorporating two targets, the Xpert® Ebola Assay may ultimately prove be more reliable than single-target assays when genomic diversity occurs. In addition, because transient viremia has been documented due to vaccination with live recombinant virus vaccines containing the EBOV GP region, the inclusion of the both targets may distinguish between natural infection and vaccination [21].

To date, there is no clear correlation between EBOV titer in specimens, number of PFU/mL and the corresponding number of viral RNA copies/mL. It is a challenge to determine actual input copy numbers, as indicated by the varying results in studies using either inactivated virus or RNA. In order to gain robust LoD data, we therefore estimated the LoD for three different specimen types; infectious virus, inactivated virus, and viral RNA. To further strengthen our results, we compared the Xpert® Ebola Assay head-to-head with two other tests that received FDA-EUA approval. In general, our data are consistent with the observation that viral RNA copies outnumber infectious virus by a factor of 100 or even 1000 to one [5].

For experiments with infectious virus, the limited number of replicates and virus concentrations precluded the use of Probit analysis. Among the different assays, LoD was estimated to be 73 copies/mL (inactivated virus), 1 PFU/mL (infectious virus), and 232 copies/mL (RNA). Virus levels in fatal cases can reach 10^8^−10^9^ genome copies/mL of serum, and for non-fatal cases on average 10^2^ lower at day 2 post onset of illness, while for non-fatal cases, RNA levels might be below the level of detection at days 1 and 2 [5, 22]. This highlights the need for a very sensitive diagnostic assay. The Xpert® Ebola Assay has a lower LoD than other currently available tests (Altona RealStar® Ebolavirus RT-PCR Kit 1.0 and BioFire Defense LLC FilmArray Biothreat-E Test®), with significant shorter turnaround time and less hands-on time (compared to the Altona assay), and lower cost and the potential to run multiple tests in parallel (compared to the Biofire assay). The lower LoD may allow cases to be detected very early after exposure and may provide better overall performance in detecting cases in field settings where sample collection can be challenging and sample degradation may occur. In addition, the use of a highly sensitive test for monitoring of infected cases may avoid premature release from isolation conditions, as well as allow analysis of other sample types such as semen during the recovery period.

The Xpert® Ebola Assay has a robust inactivation system. It has previously been shown that a 10-minute incubation period at ambient temperature using a ratio of ≥1:3 of EBOV and 30–60% guanidine chaotrope will completely inactivate EBOV [16]. The Xpert® Ebola Assay SR falls well within this range having less than a 5% dilution of 4.5 M guanidinium isothiocyante starting material. As mentioned, although EBOV RNA levels can reach the 10^8^–10^9^ genome copies/mL in serum, infectious virus levels rarely exceed 10^5^ PFU/mL blood (corresponding to 2x10^7^ RNA/mL) in EBOV positive patients [23]. As the inactivation experiments presented here were performed using EBOV isolate dilutions in culture media, additional experiments will be required to demonstrate adequate inactivation in whole blood and other clinically relevant matrices. Nevertheless, it is anticipated that the Xpert® Ebola SR should provide adequate safety by exhibiting at least a 6 log reduction of infectious EBOV.

We have shown that the Xpert® Ebola Assay also detects EBOV/Mak-C07, Yambuku-Ecran, Gabon-Ilende, and Kikwit-956210, in addition to EBOV/Yam-May. Testing of other ebolaviruses, non-ebolaviruses, bacteria, and parasites, as well as *in silico* analyses to predict the risk of cross-reactivity demonstrate that the Xpert® Ebola primer and probe sequences are specific and should not yield false positive EBOV results with samples containing these targets.

In summary, we found the Xpert® Ebola Assay to have high analytical sensitivity and specificity for the detection on EBOV in whole blood. It offers ease of use, fast turnaround time, and cloud-based monitoring of geo-positioned test results in real-time. The test has an efficient viral inactivation system, fulfills inclusivity and exclusivity criteria, and has specimen stability characteristics consistent with the need for decentralized testing. It has a specimen adequacy control to insure that samples that yield negative results are suitable for evaluation. The assay targets sequences in two EBOV genes, lowering the risk for new variants to escape detection in the test. The simplicity of the assay should enable testing in a wide variety of laboratory settings, including remote laboratories that are not capable of performing highly complex nucleic acid amplification tests, and during outbreaks where time to detection is critical. All of the nearly 9000 GeneXpert® systems deployed worldwide are capable of running this test. However, there are currently fewer than 40 systems currently available for use in EBOV endemic areas of West Africa, and it remains to be seen whether additional systems will be deployed for decentralized testing.

## Supporting Information

S1 Fig Specimen stability analysis showing Glycoprotein and Nucleoprotein Target Cycle Threshold values for specimens stored at different temperatures for up to 72 hours. Specimens were stored at 5°C (a), 25°C (b), 30°C (c), 35°C (d), and 45°C (e). EBOV Mayinga RNA at a level of 3-5x LoD was spiked into EDTA-whole blood (WB) mixed with Xpert® Ebola Sample Reagent (SR). Five replicates were tested at each condition except at time zero, where eight replicates were tested. Statistical significance of specimen stability at the conditions tested was determined by comparing cycle threshold (Ct) values of the test samples to the Ct values of the control sample (time zero) using one-way ANOVA. GP, Glycoprotein; NP, Nucleoprotein.
(TIFF)

S1 File *In silico* Exclusivity Analyses. (DOCX)

S2 File *In silico* Inclusivity Analyses. (DOCX)

S3 File nCounter Data Analysis. (DOCX)

S1 STARD Checklist Standards for Reporting of Diagnostic Accuracy (STARD) Checklist. (DOC)
