# Correction: Analytical Performance Characteristics of the Cepheid GeneXpert Ebola Assay for the Detection of Ebola Virus

**DOI:** 10.1371/journal.pone.0145896

**Published:** 2015-12-21

**Authors:** Benjamin A. Pinsky, Malaya K. Sahoo, Johanna Sandlund, Marika Kleman, Medha Kulkarni, Per Grufman, Malin Nygren, Robert Kwiatkowski, Ellen Jo Baron, Fred Tenover, Blake Denison, Russell Higuchi, Reuel Van Atta, Neil Reginald Beer, Alda Celena Carrillo, Pejman Naraghi-Arani, Chad E. Mire, Charlene Ranadheera, Allen Grolla, Nina Lagerqvist, David H. Persing

There is an error in the author byline. The first three authors, Benjamin A. Pinsky, Malaya K. Sahoo, and Johanna Sandlund, should be noted as contributing equally to this work.
