# Supplementary figures and images for: Analytical Performance Characteristics of the Cepheid GeneXpert Ebola Assay for the Detection of Ebola Virus
Source: PLoS One. 2015 Nov 12;10(11):e0142216. doi: 10.1371/journal.pone.0142216 (PMC4643052; doi:10.1371/journal.pone.0142216)

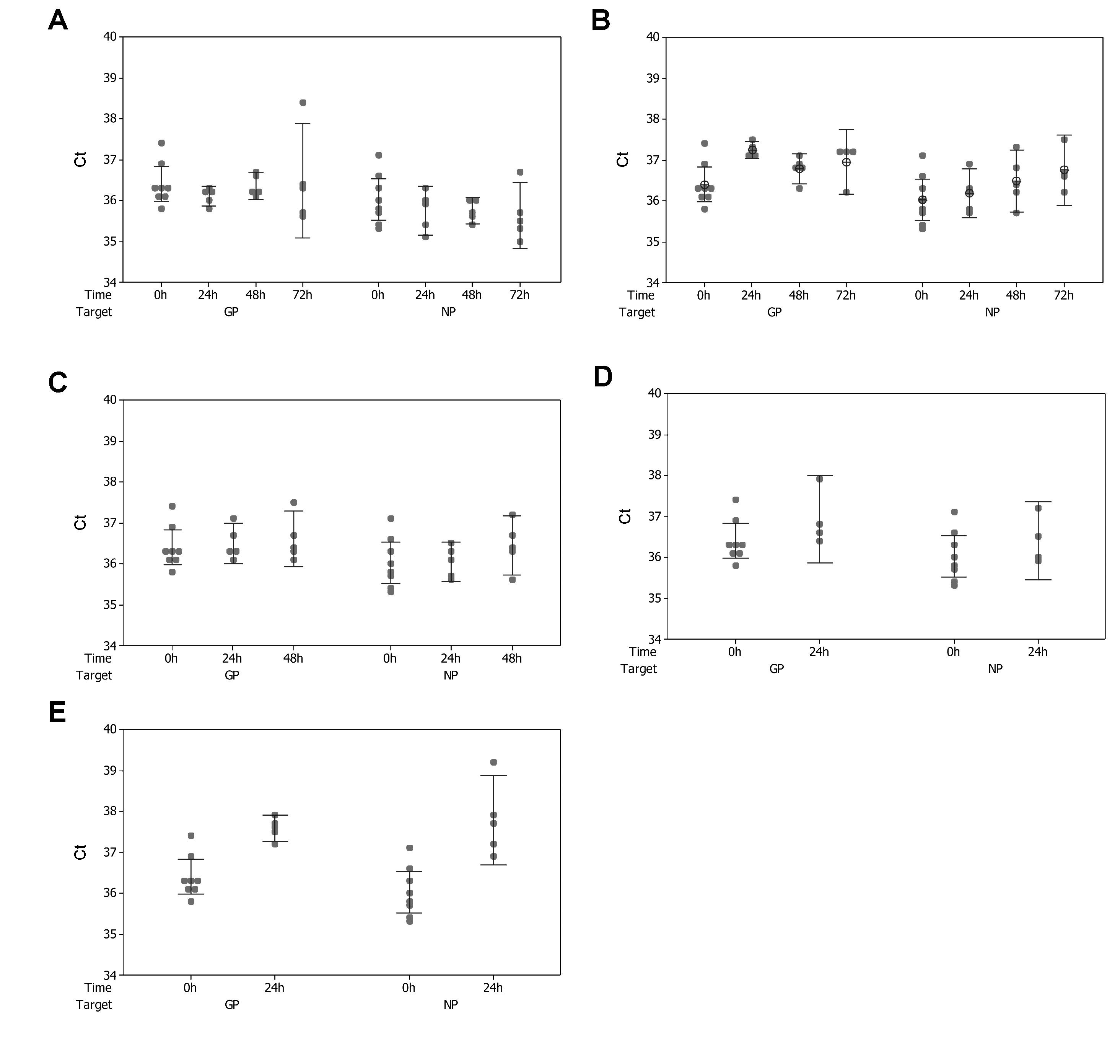

Supplement: S1 Fig — Specimens were stored at 5°C (a), 25°C (b), 30°C (c), 35°C (d), and 45°C (e). EBOV Mayinga RNA at a level of 3-5x LoD was spiked into EDTA-whole blood (WB) mixed with Xpert® Ebola Sample Reagent (SR). Five replicates were tested at each condition except at time zero, where eight replicates were tested. Statistical significance of specimen stability at the conditions tested was determined by comparing cycle threshold (Ct) values of the test samples to the Ct values of the control sample (time zero) using one-way ANOVA. GP, Glycoprotein; NP, Nucleoprotein. (TIFF) [file pone.0142216.s001.tiff]
