# Supplementary material for: Analytical Performance Characteristics of the Cepheid GeneXpert Ebola Assay for the Detection of Ebola Virus
Source: PLoS One. 2015 Nov 12;10(11):e0142216. doi: 10.1371/journal.pone.0142216 (PMC4643052; doi:10.1371/journal.pone.0142216)
Supplement: S1 File — (DOCX) [file pone.0142216.s002.docx]

**S1 Appendix**

**nCounter® Data Analysis**

Positive and negative hits were determined through MAD’s. However, because medians and MAD’s were found using the distribution all counts in a sample, lower counts in a sample with insufficient target sample concentration would show up several MAD’s above the median. Based on previous data, samples with maximum counts above 25 counts were considered valid, meaning sufficient initial concentration of nucleic acid was added, and samples with maximum counts below 25 counts were considered invalid.

If the count was over 4 MAD’s above the median and the sample’s maximum count was higher than 25 counts, then the count was considered positive, or target transcript was present. If count was over 4 MAD’s above the median but the sample’s maximum count was below 25 counts, then the count was considered negative, or target transcript was absent. In addition, if the count was below 4 MAD’s above the median then the sample was automatically considered negative.
