# Supplementary material for: Analytical Performance Characteristics of the Cepheid GeneXpert Ebola Assay for the Detection of Ebola Virus
Source: PLoS One. 2015 Nov 12;10(11):e0142216. doi: 10.1371/journal.pone.0142216 (PMC4643052; doi:10.1371/journal.pone.0142216)
Supplement: S2 File — (DOCX) [file pone.0142216.s003.docx]

**S2 Appendix**

**Inclusivity**

*In silico* analyses were performed to estimate the performance of the Xpert® Ebola Assay in detection of all EBOV variant sequences available in GenBank; from the first sequence data published in 1976 to the sequences from the current West Africa outbreak.

The two Xpert® Ebola amplicon sequences derived from EBOV Glycoprotein (GP) and Nucleoprotein (NP) genes were each submitted to BLAST. Also, all six Xpert® Ebola oligonucleotide sequences were checked individually against a local database alignment containing all EBOV sequences available in GenBank.
