# Supplementary material for: Analytical Performance Characteristics of the Cepheid GeneXpert Ebola Assay for the Detection of Ebola Virus
Source: PLoS One. 2015 Nov 12;10(11):e0142216. doi: 10.1371/journal.pone.0142216 (PMC4643052; doi:10.1371/journal.pone.0142216)
Supplement: S3 File — (DOCX) [file pone.0142216.s004.docx]

**S3 Appendix**

**Exclusivity**

*In silico* analyses were performed to estimate the risk of cross reactivity of the Xpert® Ebola Assay target oligos (Glycoprotein, GP, and Nucleoprotein, NP) towards the exclusivity disease pathogens tested here. Several approaches were used; the two Xpert® Ebola amplicon sequences derived from EBOV GP and NP genes were each submitted to BLAST. In addition, the hybridization of Xpert® Ebola oligonucleotide sequences to ebolavirus genomes versus their hybridization toward the targeted EBOV was analyzed using an *in silico* DNA analysis software from DNASoftware (VisualOMP). In addition, the Xpert® Ebola Assay control oligonucleotides (Sample Adequacy Control, SAC, and Sample Processing Control, SPC) were analyzed for potential cross reactivity to non-target sequence.
