# Supplementary material for: Analytical Performance Characteristics of the Cepheid GeneXpert Ebola Assay for the Detection of Ebola Virus
Source: PLoS One. 2015 Nov 12;10(11):e0142216. doi: 10.1371/journal.pone.0142216 (PMC4643052; doi:10.1371/journal.pone.0142216)
Supplement: S1 STARD Checklist — (DOC) [file pone.0142216.s005.doc]

# STARD checklist for reporting of studies of diagnostic accuracy

*(version January 2003)*

| **Section and Topic** | **Item**  **#** |  | **On page #** |
| --- | --- | --- | --- |
| TITLE/ABSTRACT/  KEYWORDS | 1 | **Analytical Performance Characteristics of the Cepheid GeneXpert Ebola Assay for the detection of Ebola virus**  **Background**  The recently developed Xpert® Ebola Assay is a novel nucleic acid amplification test for simplified detection of Ebola virus (EBOV) in whole blood and buccal swab samples. The assay targets sequences in two EBOV genes, lowering the risk for new variants to escape detection in the test. The objective of this report is to present analytical characteristics of the Xpert® Ebola Assay on whole blood samples.  **Methods and Findings**  This study evaluated the assay’s analytical sensitivity, analytical specificity, inclusivity and exclusivity performance in whole blood specimens. EBOV RNA, inactivated EBOV, and infectious EBOV were used as targets. The dynamic range of the assay, the inactivation of virus, and specimen stability were also evaluated.  The lower limit of detection (LoD) for the assay using inactivated virus was estimated to be 73 copies/mL (95% CI: 51-97 copies/mL). The LoD for infectious virus was estimated to be 1 plaque-forming unit/mL, and for RNA to be 232 copies/mL (95% CI 163 – 302 copies/mL). The assay correctly identified five different Ebola viruses, Yambuku-Mayinga, Makona-C07, Yambuku-Ecran, Gabon-Ilembe, and Kikwit-956210, and correctly excluded all non-EBOV isolates tested. The conditions used by Xpert® Ebola for inactivation of infectious virus reduced EBOV titer by ≥6 logs.  **Conclusion**  In summary, we found the Xpert® Ebola Assay to have high analytical sensitivity and specificity for the detection of EBOV in whole blood. It offers ease of use, fast turnaround time, and remote monitoring. The test has an efficient viral inactivation protocol, fulfills inclusivity and exclusivity criteria, and has specimen stability characteristics consistent with the need for decentralized testing. The simplicity of the assay should enable testing in a wide variety of laboratory settings, including remote laboratories that are not capable of performing highly complex nucleic acid amplification tests, and during outbreaks where time to detection is critical.  **Keywords:** Ebolavirus, molecular diagnostics |  |
| INTRODUCTION | 2 | This report describes the analytical and clinical performance characteristics of the Xpert® Ebola Assay on whole blood samples. |  |
| METHODS |  |  |  |
| *Participants* | 3 | The study population: N/A |  |
|  | 4 | Participant recruitment: N/A |  |
|  | 5 | Participant sampling: Was the study population a consecutive series of participants defined by N/A |  |
|  | 6 | Data collection: N/A |  |
| *Test methods* | 7 | The reference standard and its rationale: The Linearity and LoD results (inactivated virus) of the Xpert® Ebola Assay were compared to the results of the Altona RealStar® Ebolavirus RT-PCR Kit 1.0 (Altona Diagnostics, Hamburg, Germany) and BioFire Defense LLC FilmArray Biothreat-E Test® (BioFire Diagnostics, Salt Lake City, UT, USA). |  |
|  | 8 | Technical specifications of material and methods: The Xpert® Ebola Assay (Cepheid, Sunnyvale, CA, USA) is a novel assay for detection of EBOV from EDTA–treated whole blood obtained by venipuncture, peripheral blood derived from finger-stick, and buccal swabs. Xpert® is a fully automated RT-PCR system that integrates processing, amplification, and detection, and provides a result in 100 minutes from sample acquisition. Additionally, the system has remote monitoring and electronic reporting capability. For sample preparation, 0.1 mL whole blood or a swab to which whole blood has been allowed to absorb is transferred to the 2.5 mL Xpert® Ebola Sample Reagent (SR) bottle, which contains 4.5 M guanidinium thiocyanate. The SR buffer is transferred to the Xpert® Ebola Assay cartridge, where all steps of sample preparation, reverse transcription and multiplex, fluorescent real-time PCR occur. All reagents required for these steps are included in the cartridge, either in liquid form or in lyophilized beads. Nucleic acids are isolated and purified, concentrated onto a small glass fiber column integrated into the base of the cartridge, and washed and eluted prior to combining with the RT-PCR reagents. |  |
|  | 9 | Definition of and rationale for the units, cut-offs and/or categories of the results of the index tests and the reference standard: For Linearity and LoD testing, the results are reported in copy number (virus or RNA) per mL, which is the appropriate way to detect this specimen type. For Inclusivity and Exclusivity testing, the results are reported in the appropriate unit for each organism. |  |
|  | 10 | The number, training and expertise of the persons executing and reading the index tests and the reference standard: LoD and Linearity (inactivated virus): 2 (MD/PhD, and PhD). LoD (RNA): 2 (PhD, BS). LoD (active virus): 2 (PhDs) |  |
|  | 11 | Whether or not the readers of the index tests and reference standard were blind (masked) to the results of the other test and describe any other clinical information available to the readers: Not blinded |  |
| *Statistical methods* | 12 | Methods for calculating or comparing measures of diagnostic accuracy, and the statistical methods used to quantify uncertainty (e.g. 95% confidence intervals): Oridnary least squares regression and Probit analysis. |  |
|  | 13 | Methods for calculating test reproducibility, if done: Linearity testing in replicates of 3. LoD testing in replicates of 20. |  |
| RESULTS |  |  |  |
| *Participants* | 14 | When study was performed, including beginning and end dates of recruitment: N/A |  |
|  | 15 | Clinical and demographic characteristics of the study population (at least information on age, gender, spectrum of presenting symptoms): N/A |  |
|  | 16 | The number of participants satisfying the criteria for inclusion who did or did not undergo the index tests and/or the reference standard; describe why participants failed to undergo either test (a flow diagram is strongly recommended): N/A |  |
| *Test results* | 17 | Time-interval between the index tests and the reference standard, and any treatment administered in between: N/A |  |
|  | 18 | Distribution of severity of disease (define criteria) in those with the target condition; other diagnoses in participants without the target condition: N/A |  |
|  | 19 | A cross tabulation of the results of the index tests (including indeterminate and missing results) by the results of the reference standard; for continuous results, the distribution of the test results by the results of the reference standard. N/A |  |
|  | 20 | Any adverse events from performing the index tests or the reference standard: N/A |  |
| *Estimates* | 21 | Estimates of diagnostic accuracy and measures of statistical uncertainty (e.g. 95% confidence intervals). LoD (inactivated virus): 73 copies/mL (95% CI: 51-97 copies/mL). LoD (RNA): 232 copies/mL (95% CI 163- 302 copies/mL). LoD (infectious virus: 1.0 PFU/mL. |  |
|  | 22 | How indeterminate results, missing data and outliers of the index tests were handled: In LoD testing (inactivated virus), one cartridge had a manufacturing error and the replicate was therefore removed. |  |
|  | 23 | Estimates of variability of diagnostic accuracy between subgroups of participants, readers or centers, if done: N/A. |  |
|  | 24 | Estimates of test reproducibility, if done: Linearity testing in replicates of 3. LoD testing in replicates of 20. |  |
| DISCUSSION | 25 | Discuss the clinical applicability of the study findings: The simplicity of the assay should enable testing in a wide variety of laboratory settings, including remote laboratories that are not capable of performing highly complex nucleic acid amplification tests, and during outbreaks where time to detection is critical. We found the Xpert® Ebola Assay to have high analytical sensitivity and specificity for the detection on EBOV in whole blood. It offers ease of use, fast turnaround time, and cloud-based monitoring of geo-positioned test results in real-time. The test has an efficient viral inactivation system, fulfils inclusivity and exclusivity criteria, and has specimen stability characteristics consistent with the need for decentralized testing. It has a specimen adequacy control to insure that samples that yield negative results are suitable for evaluation. The assay targets sequences in two EBOV genes, lowering the risk for new variants to escape detection in the test. |  |
